# Upper limb training for young children with unilateral cerebral palsy using video coaching: An explorative retrospective clinical study

**DOI:** 10.1111/1440-1630.70008

**Published:** 2025-03-24

**Authors:** Anke P. M. Verhaegh, Steven Teerenstra, Maria W. G. Nijhuis‐van der Sanden, Pauline B. M. Aarts, Michèl A. A. P. Willemsen, Brenda E. Groen

**Affiliations:** ^1^ Department of Paediatric Rehabilitation Sint Maartenskliniek Nijmegen The Netherlands; ^2^ IQ Healthcare, Research Institute for Health Sciences Radboud University Medical Center Nijmegen The Netherlands; ^3^ Department for Health Evidence, Section Biostatistics, Research Institute for Health Sciences Radboud University Medical Center Nijmegen The Netherlands; ^4^ Department of Rehabilitation, Donders Institute for Brain, Cognition and Behaviour Radboud University Medical Center Nijmegen The Netherlands; ^5^ Department of Research Sint Maartenskliniek Nijmegen The Netherlands; ^6^ Department of Paediatric Neurology Amalia Children's Hospital, Radboud University Medical Center Nijmegen The Netherlands; ^7^ Donders Institute for Brain, Cognition and Behavior Radboud University Medical Center Nijmegen The Netherlands

**Keywords:** early intervention, home training program, occupational therapy, unilateral cerebral palsy, upper limbs, video coaching

## Abstract

**Introduction:**

Young children with unilateral cerebral palsy (CP) received a home‐based training program using video coaching for parents. The primary aim of our study was to evaluate the effectiveness of unilateral training on the use of the affected arm and hand during bimanual activities and to explore factors that affect treatment response. Secondary, we evaluated whether effects were retained after an 8‐week break, and if data were available, we explored the effects of a second uni‐ or bimanual training block. Furthermore, adherence was evaluated.

**Methods:**

Explorative retrospective clinical study evaluating the effectiveness of the first 8‐week training block on the (Mini‐) Assisting Hand Assessment ((Mini‐) AHA) unit score in 81 children aged 8–36 months. Pre‐ and post‐intervention (T0–T1) and 8‐week follow‐up measurements (T2) were evaluated, and factors influencing treatment response were explored, using linear mixed models (LMM). Additionally, effects of a second training block were explored in 31 of the original 81 children, contingent upon data availability, with T3–T4 measurements included. Adherence, measured as percentage of treatment duration, was explored.

**Results:**

Mini‐AHA and AHA unit scores significantly improved between T0 and T1, but did not change between T1 and T2. In children aged 18 months and older, baseline AHA scores were related to change scores. In children aged <18 months, no predictors of treatment response were identified. LMM showed significant improvement between T1–T3 and T1–T4 in Mini‐AHA scores in children with a second training block. Adherence rates were 85% in the first and 81% in the second block.

**Conclusions:**

Our data suggest that upper limb training using video coaching can improve hand use in infants and toddlers with unilateral CP, with retained effects after an 8‐week break and further enhancement following a second training block. Individual results differed, and controlled studies are needed to strengthen the evidence. High adherence rates suggest the program's feasibility.

**Consumer and Community Involvement Statement:**

There was no direct consumer and community involvement in the study design.

**PLAIN LANGUAGE SUMMARY:**

Cerebral palsy is caused by a brain injury around birth and is the most common physical disability in children, affecting their movement. Children with one side of the body affected often use that side less, making daily activities harder. Training the affected arm in the first 2 years of life is important because the brain is still very adaptable. In our study, we evaluated a home‐based training program for young children with cerebral palsy, with blocks of 8 weeks of therapy using video coaching for parents. We looked at how well the first training block improved the use of the affected arm and hand. We also looked at whether the effects lasted after an 8‐week break and whether a second training block further improved hand use. Lastly, we looked at how well families continued to train. We found an improvement of the use of the affected hand after the first training block. Children older than 18 months with poorer hand use at the start made more progress, while especially children younger than 18 months demonstrated further improvement after the second training block. Most parents and children were able to continue the training program using video coaching. Early upper limb home‐based training with video coaching can help young children with cerebral palsy to improve the use of their affected arm and hand. Video coaching seems effective to motivate parents to continue with the program. Individual results varied. There is a need for larger studies.

**Key Points for Occupational Therapy**

- The program seems effective; children aged ≥18 months with lower baseline scores benefit more.
- Home‐based early upper limb training using video coaching seems feasible to improve affected hand use.
- Video coaching in occupational therapy practice may contribute to future accessibility to specialised interventions.

## 1 INTRODUCTION

Since 2013, the international birth prevalence of children with cerebral palsy declined from an estimate of 2.1 per 1000 live births, to 1.6 per 1000 live births (in high‐income countries) (McIntyre et al., 2022; Oskoui et al., 2013). Unilateral cerebral palsy (CP) accounts for about 40% of children with CP (Wu et al., 2006). Many children with unilateral CP disregard the use of the affected upper limb (referred to as ‘developmental disregard’) when they discover that it is more efficient to perform activities using the less affected hand (Hoare et al., 2019; Zielinski et al., 2014). This may lead to a diminished bimanual task performance and reduced independence in daily life (Hoare & Greaves, 2017).

Infants at high risk of developing CP should start early with CP upper limb interventions. This is important due to elevated levels of brain plasticity in the first 2 years of life, presenting a window of opportunity for optimal brain development and maximising functional outcomes (McIntyre et al., 2011; Morgan et al., 2021). Additionally, it is recognised that in children with unilateral CP, the use of the affected hand primarily develops during the early preschool period, and bimanual performance stabilises around 7 years of age (Eliasson et al., 2022). Moreover, research indicates that a higher level of performance at the age of 18 months predicts faster rates and higher limits of future bimanual performance (Nordstrand et al., 2016).

There is strong evidence for the efficacy of training‐based upper limb interventions for older children with CP, including constraint‐induced movement therapy and bimanual training (Hoare et al., 2019; Novak et al., 2020). For infants and toddlers, however, evidence regarding efficacy of early upper limb intervention programs is still limited (Mailleux et al., 2021; Morgan et al., 2016). Only three RCTs (Chamudot et al., 2018; Eliasson et al., 2018; Hwang & Kwon, 2020) have been performed to support the efficacy of home‐based, high‐intensity modified Constraint Induced Movement Therapy (mCIMT) and Bimanual Training (BiT) to improve affected upper limb function and bimanual performance in infants (3–36 months of age) with unilateral CP. Additionally, a retrospective study (Nordstrand et al., 2015) revealed that participation in a baby‐CIMT program during the first year of life increases the chance of achieving better bimanual activity performance at 2 years of age, suggesting long‐term effects. These previous studies (Chamudot et al., 2018; Eliasson et al., 2018) demonstrate significant interindividual variability in treatment response. For older children (>2 years of age), it has been shown that children with lower baseline function tend to show larger gains (Sakzewski et al., 2014). For infants, predictors of treatment response such as baseline function, age at the start of the intervention, gestational age, birth weight, and training intensity were not correlated with the effects of intervention (Chamudot et al., 2018; Eliasson et al., 2018). This may be due to small sample sizes (*n* < 20) in these infant studies.

Considering the demand on parents to provide training to their child (Beckers et al., 2021), in 2013, we developed an early upper limb home‐based training program with (if applicable repeated) 8‐week blocks of mCIMT or BiT, using a video‐coaching approach (Verhaegh et al., 2022) to enable participation in the program for parents and children not living nearby our rehabilitation centre. Moreover, the online video coaching increases flexibility for parents compared with scheduling home visits or online web‐based appointments with the therapist. In our approach, parents uploaded their videos of the home training sessions weekly, and coaching was conducted remotely through written exchanges between parents and the therapist.

In a previous study (Verhaegh et al., 2022), interviews with 13 parents who participated in our upper limb training program revealed that video coaching increased their competence in providing the training and it motivated parents to continue with the program. In addition, parents appreciated the video coaching as it increases the flexibility in planning the training sessions: there is no need for travelling to the rehabilitation centre and no need for planning home visits by a therapist. Furthermore, parents appreciated the block‐based approach; knowing that the intensive training block would end after 8 weeks stimulated parents to persevere in providing the training until the break. Parents' experiences indicate that this home‐based training program using video coaching is feasible; however, its effectiveness and actual adherence still need to be investigated.

The primary aim of this explorative retrospective clinical study is to evaluate the effectiveness of the first home based mCIMT training block using a video‐coaching approach for infants and toddlers (8–36 months of age) with unilateral CP on the use of the affected arm and hand during bimanual activity performance and to explore factors that may affect treatment response. The secondary aims of our study included: evaluating whether the effects are retained after an 8‐week break; evaluating the impact of a second mCIMT or BiT training block on use of the affected arm and hand during bimanual activity performance (if data of a second training block were available); and evaluating the adherence to the training program using registered training duration.

In this study, we evaluate the effectiveness of our home‐based program using a video‐coaching approach for parents in everyday clinical settings to ascertain whether our results are consistent with those of previous controlled trials using home‐visits.

## 2 METHODS

### 2.1 Design

This study was an explorative retrospective clinical study. Pre‐ and post‐measurements were used to evaluate a home‐based upper limb training program with an 8 weeks block of intensive training (mCIMT) using a video‐coaching approach. Measurements were performed before (T0) and after (T1) the intensive upper limb training and at follow‐up after an 8 weeks break (T2). In a subgroup of children, data of a second training block were available (mCIMT or BiT), with a measurement after the second 8 weeks training block (T3) and a second follow‐up after an 8 weeks break (T4).

Due to the nature of the data collection process, which involved sampling of pre‐existing data from patient files by clinicians, the anonymous handling of information along with informed consent from the parents, the study was exempted from further ethical approval by the medical ethical review board (METC‐Oost Nederland, protocol reference number: 2020‐6185) in 2020 in accordance with the Dutch Medical Research Involving Humans Acts. Approval for the use of file data was granted by the participating rehabilitation centres.

### 2.2 Positionality statement

The first author (A.V.) of this paper is an experienced occupational therapist (MSc) with expertise in CIMT and BiT interventions for children with CP. In this study, she acted as both researcher and occupational therapist and coached some of the families. S.T., an experienced statistician, provided independent statistical advice and carried out some analysis without being involved in the children's treatment. M.N., P.A., M.W. and B.G. were senior researchers and supervisors with extensive clinical and research experience in paediatric rehabilitation, CIMT and BiT interventions and rehabilitation science. None were involved in the treatment of the children, although M.W. referred some children as a paediatric neurologist.

### 2.3 Participants

In five paediatric rehabilitation centres in the Netherlands, data were collected between 2014 and 2021 from children who participated in at least one early mCIMT training block using video coaching, provided that their parents had signed informed consent to use the clinical data for study purposes. Inclusion criteria were clinical signs of unilateral CP or at risk of developing unilateral CP as diagnosed by the referring physician (preferably confirmed by cerebral ultrasound studies or magnetic resonance imaging [MRI]) and a (corrected) age between 8 and 36 months during the training program. Exclusion criteria were clinical signs of bilateral CP or other neurological conditions as diagnosed by the referring physician or the therapist deviating from the intended training protocol.

### 2.4 Procedure

In 2013, three therapists of the Sint Maartenskliniek (A.V., P.A., and I.T.) adapted their group based on mCIMT‐BiT model for children from the age of 2.5 years (pirate group intervention) (Aarts et al., 2012) to a version suitable for infants and toddlers (8–36 months). A video‐coaching approach was incorporated to facilitate parental participation. Following the implementation in the Sint Maartenskliniek in 2014, therapists at four other rehabilitation centres in the Netherlands were trained upon their own request between 2016 and 2019 to deliver the intervention in clinical practice. All of these centres were familiar with delivering mCIMT‐BiT interventions. The training consisted of a 1‐day session, which included background information, case studies, and practice sessions involving reflections on videos of multiple home‐training sessions. After training, we remained available for questions and feedback. Annually, we met therapists from implementing centres to discuss their experiences with the training, addressing any questions about the intervention or video coaching. It was advocated in the educational course to evaluate the intervention every 8 weeks using the (Mini‐) AHA. Additionally, therapists were requested to ask informed consent from parents to use anonymous clinical data for future research purposes. In 2020, the decision was made to design the current study.

### 2.5 Intervention

The intervention was designed as an intensive home‐based goal directed upper limb training program using video coaching of the parents. For a detailed description of our training program, see Verhaegh et al. (2022). During the first visit at the rehabilitation centre, the focus was on connecting with parents by listening to their story and exploring their needs and goals. If parents decided to participate in the training program, they received information from the occupational therapist about the goal of the intervention (improving specific unimanual capacities). Additionally, they were provided with a box containing toys and instructions on how and when to conduct the training at home tailored to their own and their child's individual needs and context. The intensive training model started with an 8‐week block of mCIMT aimed at improving unimanual capacities of the affected upper limb. The training consisted of 30 minutes of training daily, 7 days per week, provided by parents in the home situation, followed by an 8‐week break. During the evaluation at the rehabilitation centre following this break, a decision was made in collaboration with the parents on whether to proceed immediately with a second training block. If continued, the choice was between a second mCIMT or a BiT block. The BiT block places emphasis on repetitive performance of goal‐related, two‐handed activities that elicit specific bimanual actions. The decision on the type of intervention was informed by several factors: assessment outcomes (predicting whether improving unimanual capacities first enhances bimanual performance or if direct focus on bimanual skill strategies is more beneficial), the developmental stage of the child (aligning developmental stages of play with practice type to sustain child motivation and engagement) and parental preferences (unimanual training can be easier to conduct, while a bimanual approach can be more enjoyable).

Remote asynchronous online video coaching by the occupational therapist supported parents in delivering the training to their child at home. Each week, parents uploaded videos of the home training sessions and added their written comments (i.e., remarks, questions, concerns, and reflections) to the videos on a secure website. The therapist from the rehabilitation centre provided written feedback on the videos and shared their thoughts and suggestions. During the video coaching, the therapist took on different roles to provide timely information on observations regarding hand function development, stages of play, and toy and play suggestions. They guided and coached parents to gain insights and identify the training opportunities that worked best for their child and family (Verhaegh et al., 2022).

In the Netherlands, every infant experiencing developmental delays receives paediatric physical therapy at home. The intensity of this therapy varies and continued throughout the 8‐week training block and the subsequent 8‐week break. The primary focus of the physical therapist is on (gross motor) milestone development. Collaboration between the physical therapist and the therapist from the rehabilitation centre primarily takes place through communication via the secure website, email or telephone.

### 2.6 Data collection

Measurements were registered in electronic patient records as part of clinical practice by a total of 11 therapists from five different rehabilitation centres. Anonymous data were shared with the researcher in 2020–2021 using data registration forms developed for the study. At baseline, data on (corrected) age in months, gender, diagnosis, results of cerebral imaging studies, (more) affected side, Mini‐MACS classification, and (Mini‐) AHA sum and unit score were collected. Furthermore, therapists registered type of training and total training duration for each training block, as well as the (Mini‐) AHA sum and unit scores for each measurement. Completed data registration forms were sent to the first author (A.V.). Informed consents were signed and stored within the rehabilitation centres involved in the treatment of the child.

### 2.7 Measures

#### 2.7.1 Baseline characteristics

Brain lesions were classified into five categories (see Table 1). The Mini‐MACS was used to classify how the child uses its hands when handling objects in daily live activities (Eliasson et al., 2017).

**TABLE 1:** Participant demographic and baseline (T0) characteristics.

	Children with, or at risk of unilateral CP (*n* = 81)
(Corrected) age in months, mean (SD), range	14.6 (6.0), 8–35
Gender, M/F, *n*	44/37
Preterm (GA < 37 weeks)/term, *n*	20/61
Affected side left/right, *n*	44/37
**Neuroimaging**	
PAIS/PVHI/non‐specific/normal MRI/brain malformation/imaging not available, *n*	37/31/3/1/1/8
**Mini AHA unit score (*n* = 59): Mean (SD)**	37.7 (21.0)
**AHA unit score (*n* = 22): Mean (SD)**	51.1 (22.3)
Mini MACS I/ II/ III/IV/V, *n*	20/26/29/5/0
Ability to grasp yes/no, *n*	39/42

*Note*: Corrected age was calculated for infants born <37 weeks.

Abbreviations: (Mini‐) AHA, (Mini‐) Assisting Hand Assessment; Mini‐MACS, Mini‐Manual Abilitiy Classification System (level at initial visit or ≥12 months of age); MRI, magnetic resonance imaging; PAIS, perinatal arterial ischaemic stroke; PVHI, periventricular haemorrhagic infarction.

#### 2.7.2 Outcome measures

To measure how effectively children use the affected upper limb in bimanual activities, trained and certified therapists scored the Mini‐Assisting Hand Assessment (Mini‐AHA) for children aged 8–18 months (Greaves et al., 2013) and the Kids‐AHA for children aged 18 months and older (Krumlinde‐Sundholm & Eliasson, 2003) at each measurement point (T0–T4). These therapists were also involved in the treatment of the child.

#### 2.7.3 Assisting hand assessment (AHA)

The Assisting Hand Assessment (AHA) is a criterion‐referenced tool used to describe and measure how effectively a child (18 months to 12 years old) with unilateral upper limb impairment uses the affected arm and hand for bimanual tasks (Krumlinde‐Sundholm & Eliasson, 2003). The AHA (version 4.4) has strong psychometric properties (Holmefur et al., 2009; Krumlinde‐Sundholm et al., 2007). A low association between age and AHA score indicates that a higher score reflects increased ability rather than age‐dependent development (Krumlinde‐Sundholm et al., 2007). The smallest detectable difference (SDD) of the AHA is 4 raw score points, equivalent to 5 AHA units (Krumlinde‐Sundholm, 2012). The Kids‐AHA (version 5.0) includes 20 items scored on a 4‐point rating scale providing a raw score of 20 to 80, which can be converted into AHA units ranging from 0 to 100. Scoring is based on video recordings of the child's spontaneous handling of toys requiring bimanual hand use (Holmefur & Krumlinde‐Sundholm, 2016). The Kids‐AHA 5.0 is expected to have similar reliability to the AHA 4.4, though this has not yet been investigated (Holmefur & Krumlinde‐Sundholm, 2016).

In our study, the Kids‐AHA version 5.0 scoring criteria were used to score the videos, or scores of the 4.4 version were converted to version 5.0 using the score conversion table (Holmefur & Krumlinde‐Sundholm, 2016).

#### 2.7.4 Mini‐assisting hand assessment (Mini‐AHA)

The Mini‐AHA is used for children aged 8–18 months. Like the AHA, it scores 20 items on a 4‐point scale, with higher scores indicating better ability (Greaves et al., 2013). Sum scores (20–80) are converted into unit scores (0–100). Typically developing children in this age range achieve maximum scores on each item, with age and Mini‐AHA scores showing a negligible relationship (Greaves et al., 2013). Test–retest, inter‐ and intra‐rater reliability, and responsiveness to change have not yet been studied.

#### 2.7.5 Adherence

Adherence to the training program was evaluated by training duration. Parents were asked to fill out a digital time registration form, recording the minutes of training per day, on a secure website.

### 2.8 Data analysis

Descriptive statistics were used to describe baseline data. For the analysis regarding the primary research question, a linear mixed model analysis (LMM) was conducted on the delta (Mini‐) AHA scores of the first training block using the ∆T0–T1 and ∆T0–T2 as repeated measures within child. A LMM was applied to account for the correlation due repeated measurements within a child and handle possible missing data (under the missing‐at‐random assumption). The intercept (regression coefficient) expressed the estimated ∆T0–T1 score. A dummy variable for the additional effect for ∆T1–T2 (i.e., ∆T0–T2 minus ∆T0–T1) was included in the model as fixed effect. An unstructured covariance type was used and Satterthwaite's degrees of freedom for the fixed effects.

To check for redundancy among the baseline (T0) variables, correlation between Mini‐MACS level, baseline (Mini‐) AHA unit score, age, and gestational age was calculated to assess collinearity. To assess which of the remaining factors at baseline affected treatment response, those factors that correlated with ∆T0–T1 (Mini‐) AHA were included as covariates in the model. If correlated with ∆T0–T1 (Mini‐) AHA training duration was included as covariate in the model. Model assumptions (residuals being normally distributed and uncorrelated to predicted values) were assessed using residuals versus predicted plots. Akaike information criterion (AIC) was used to compare different possible models. Formal testing of nested models was performed by comparing the −2 log likelihood to a chi‐square distribution with the difference in fixed effect parameters. The model with the fewest covariates and similar fit to the model with all covariates was selected.

The same procedure as described above was followed to analyse the effectiveness of a second training block (mCIMT or BiT). Extra dummy variables for ∆T1–T3 and ∆T1–T4 were included in the model as fixed effect. The ∆T1–T3 assessed the added effect of the second training compared with the direct effect of the first training (i.e., ∆T0–T1) and similarly ∆T1–T4 assessed the effect after the second 8‐week break compared to the first training (i.e., ∆T0–T1). As scores of the Kids‐AHA and Mini‐AHA cannot yet be compared, we performed separate analyses for both measures. Analyses were carried out using IBM SPSS Statistics software (version 27).

Adherence was calculated as the average training duration in hours as a percentage of the total planned treatment duration in 8 weeks (28 hours).

## 3 RESULTS

**FIGURE 1:**
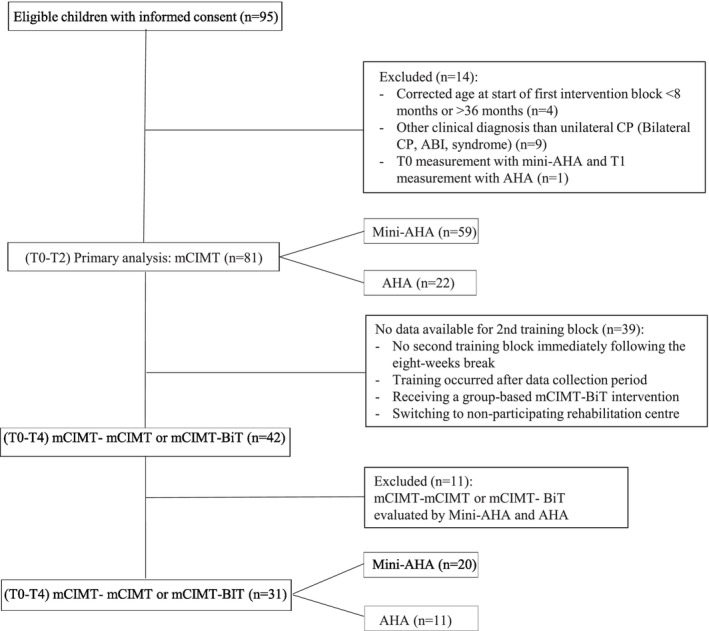
Flowchart of the inclusion and exclusion of participants.

Data from 95 children who participated in the early upper limb home‐based training program were collected (see Figure 1). However, data from 14 children were excluded: four children were younger than 8 months, six children were diagnosed by the referring physician as bilateral CP, two children had a traumatic brain injury (TBI), one child had heart failure, and one child underwent Mini‐AHA assessment at T0 and AHA at T1. It was decided not to exclude data from children classified by the therapist as Mini‐MACS IV (mean (corrected) age at T0; 13.6 months), as it has been suggested that the Mini‐MACS is less stable in early age (Klevberg et al., 2018). Additionally, these children were diagnosed as unilateral CP by the referring physician, as was confirmed with brain imaging. Therefore, data from 81 children (59 children measured with the Mini‐AHA and 22 children measured with the AHA) were included in the (primary) analyses of the first training block.

Data from a second training block were available for 42 of the 81 children included in the analyses of the first training block (see Figure 1). Reasons for non‐availability of data of a second training block included: no second training block immediately following the 8‐week break, the second training block occurring after the data collection period, receiving a group‐based mCIMT‐BiT intervention after the first block, and switching to another rehabilitation centre. In addition, data from 11 children were excluded due to the evaluation of the first training block with the Mini‐AHA and the second training block with the AHA. Therefore, data from 31 children (20 children measured with the Mini‐AHA, and 11 children measured with the AHA) were included in the analyses for the second training block (see Figure 1).

### 3.1 Effectiveness of the first block of mCIMT (T0–T2)

To evaluate the effectiveness of the first block of mCIMT, longitudinal analyses over T0 to T2 were conducted for the Mini‐AHA (*n* = 59) and the AHA (*n* = 22), separately. There were two missing values on T2 of the Mini‐AHA, one missing value on T1 and three missing values on T2 of the AHA with mostly reasons unrelated to the outcome. LMM analysis revealed an improvement in Mini‐AHA unit score (*P* < 0.05) and AHA unit score (*P* < 0.05) between T0 and T1. Between T1 and T2, no significant changes in the Mini‐AHA (*P* > 0.05) or AHA (*P* > 0.05) were found (see Table 2).

**TABLE 2:** T0–T2 (mCIMT) Mini‐AHA and AHA without covariates.

Outcome measure	Time	Estimate (SE)	95% CI	*P*
Mini‐AHA (*n* = 59)	∆T0–T1	8.4 (1.2)	6.0 to 10.9	<0.001
	∆T1–T2	2.3 (1.4)	−0.5 to 5.0	0.108
AHA (*n* = 22)	∆T0–T1	4.9 (1.2)	2.4 to 7.4	0.001
	∆T1–T2	1.0 (0.87)	−0.8 to 2.8	0.259

The Mini‐MACS showed moderate correlation with baseline Mini‐AHA unit score (*r* = 0.57). Baseline Mini‐AHA, age, gestational age and training intensity did not correlate with ∆T0–T1 Mini‐AHA. Therefore, no covariables were included in the LMM model for outcomes on the Mini‐AHA. Regarding the outcomes on the AHA, baseline AHA and Mini‐MACS were highly correlated (*r* = 0.76), and age and gestational age showed moderate correlation (*r* = 0.51). Age and training duration did not correlate with ∆T0–T1 AHA, but baseline AHA moderately correlated with ∆T0–T1 AHA (*r* = 0.37). Consequently, only baseline AHA was considered as a covariate in the LMM model. Formal testing of this model resulted in a statistically significantly improved fit (estimated effect: −0.12 [SE 0.05]). A lower AHA at baseline was associated with a larger positive change.

**FIGURE 2:**
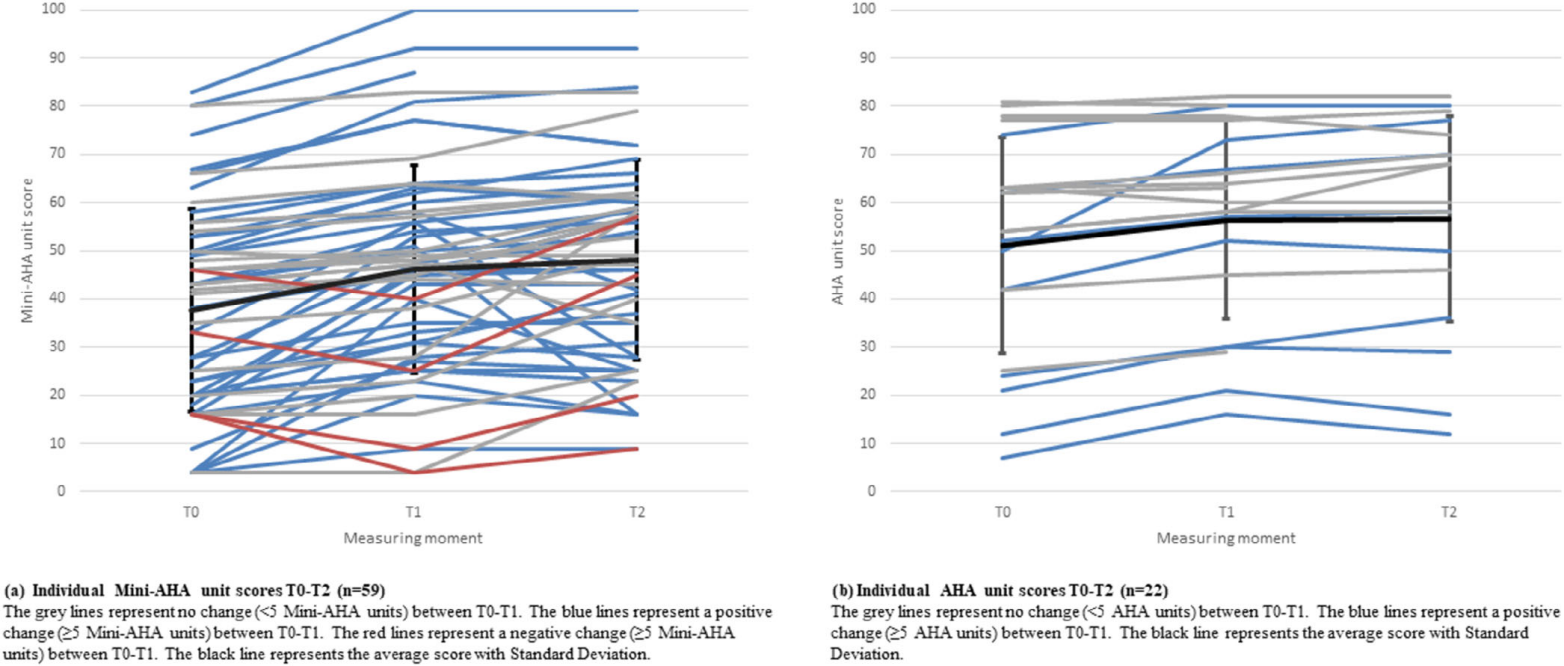
(a) Individual Mini‐AHA unit scores T0–T2 (*n* = 59). The grey lines represent no change (<5 Mini‐AHA units) between T0 and T1. The blue lines represent a positive change (≥5 Mini‐AHA units) between T0 and T1. The red lines represent a negative change (≥5 Mini‐AHA units) between T0 and T1. The black line represents the average score with standard deviation. (b) Individual AHA unit score T0–T2 (*n* = 22). The grey lines represent no change (>5 AHA units) between T0 and T1. The blue lines represent a positive change (≥5 AHA units) between T0 and T1. The black line represents the average score with standard deviation.

Interindividual variations in change scores of ≥5 units on the (Mini‐) AHA in response to the first training block were observed, as is visualised in Figure 2a,b. For the Mini‐AHA, a positive change of ≥5 units between T0 and T1 (immediately after the first training block) was observed in 36 children, while a negative change of ≥5 units was observed in four children. For the AHA, a positive change of ≥5 units between T0 and T1 was observed in nine children, while no negative change of ≥5 units was observed (see Table 3).

**TABLE 3:** Individual change in (Mini‐) AHA unit scores in children with one training block (T0–T2) (*n* = 81).

Outcome measure		Positive change ≥5 units (*n*)	Negative change ≥5 units (*n*)	Missing (*n*)
Mini‐AHA (*n* = 59)	ΔT0–T1	36	4	0
	ΔT0–T2	43	5	2
AHA (*n* = 22)	ΔT0–T1	9	0	1
	ΔT0–T2	11	0	3

### 3.2 Effectiveness of the second block of intensive upper limb training mCIMT or BiT (T1–T3, T1–T4)

To evaluate the effectiveness of a second training block (mCIMT or BiT), the added effects of the second training block compared with the effects of the first training block were assessed. As data from 31 children could be included in the analyses of this second training block, longitudinal sub‐analyses were conducted on these data for the Mini‐AHA (*n* = 20) and for the AHA (*n* = 11), separately. Additionally, there were three missing values on T4 of the Mini‐AHA and one missing value on T1 of the AHA, again mostly for unrelated reasons.

Between T1 and T3, an improvement in Mini‐AHA unit score (*P* < 0.05) was found. For the AHA, a non‐significant trend for change in AHA unit scores was found (*P* > 0.05). Between T1 and T4, an improvement in Mini‐AHA unit scores was found (*P* < 0.05). On the AHA, a non‐significant trend for change in AHA unit scores was found (*P* > 0.05). In order to compare whether the subgroup showed results similar to the total group (*n* = 59 Mini‐AHA; *n* = 22 AHA), we repeated the analysis for the subgroup across T0–T4 (see Table 4).

**TABLE 4:** T0–T4 (mCIMT‐mCIMT and mCIMT‐BiT) Mini‐AHA and AHA without covariates.

Outcome measure	Time	Estimate (SE)	95% CI	*P*
Mini‐AHA (*n* = 20)	∆T0–T1	8.2 (2.8)	2.2 to 14.1	0.010
	∆T1–T2	4.4 (2.7)	−1.3 to 10.1	0.121
	∆T1–T3	11.0 (2.5)	5.7 to 16.2	<0.001
	∆T1–T4	14.3 (3.0)	7.9 to 20.6	<0.001
AHA (*n* = 11)	∆T0–T1	5.6 (0.9)	3.5 to 7.6	<0.001
	∆T1–T2	0.5 (1.3)	−2.4 to 3.4	0.698
	∆T1–T3	4.0 (1.8)	−0.1 to 8.0	0.053
	∆T1–T4	3.6 (1.7)	−0.06 to 7.3	0.053

**FIGURE 3:**
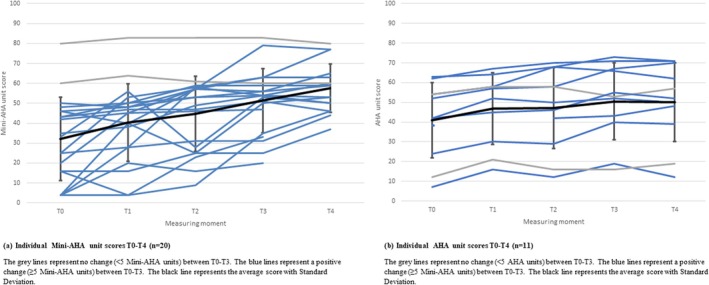
(a) Individual Mini‐AHA unit scores T0–T4 (*n* = 20). The grey lines represent no change (<5 Mini‐AHA units) between T0 and T3. The blue lines represent a positive change (≥5 Mini‐AHA units) between T0 and T3. The black line represents the average score with standard deviation. (b) Individual AHA unit scores T0–T4 (*n* = 11). The grey lines represent no change (<5 Mini‐AHA units) between T0 and T3. The blue lines represent a positive change (≥5 Mini‐AHA units) between T0 and T3. The black line represents the average score with standard deviation.

Interindividual variation in change scores of ≥5 units on the (Mini‐) AHA in response to the second training block was observed, as visualised in Figure 3a,b. For the Mini‐AHA (*n* = 20), a positive change of ≥5 units between T0 and T1 (immediately after the first training block) was observed in 7 children and between T0 and T3 (immediately after the second training block) in 18 children. For the AHA (*n* = 11), a positive change of ≥5 units between T0 and T1 was observed in six children and between T0 and T3 in nine children (see Table 5).

**TABLE 5:** Individual change in (Mini‐) AHA unit scores in children with two training blocks (T0–T4) (*n* = 31).

Outcome measure		Positive change ≥5 units (*n*)	Negative change ≥5 units (*n*)	Missing (*n*)
Mini‐AHA (*n* = 20)	ΔT0–T1	7	2	0
	ΔT0–T2	15	2	0
	ΔT0–T3	18	0	0
	ΔT0–T4	14	0	3
AHA (*n* = 11)	ΔT0–T1	6	0	1
	ΔT0–T2	7	0	0
	ΔT0–T3	9	0	0
	ΔT0–T4	10	0	0

### 3.3 Adherence

In 28 out of the 79 children for one or both of the training blocks, the time registration form was not (completely) filled out by the parents. For these children, the therapist estimated the total training duration based on their perception of adherence during the training block and evaluations with parents after its completion. In two cases, no estimation of the time registration was provided. During the first training block (*n* = 79), the average received training duration was 85% (24 hours, SD = 8 hours), while during the second training block (*n* = 31), it was 81% (23 hours, SD = 6 hours).

## 4 DISCUSSION

In this explorative retrospective clinical study, an improvement was observed in affected hand use during bimanual activity performance, particularly following the first mCIMT training block using video coaching. No changes in affected hand use were observed after an 8‐week break suggesting that effects were retained. A lower AHA score at baseline was associated with a larger positive change in affected hand use during bimanual activity performance only in children ≥18 months. Sub‐analysis suggested additional improvements in affected hand use during bimanual activity performance after a second training block, but only significant for children <18 months of age. High adherence rates were found during both training blocks, indicating the feasibility of the training program using video coaching, for most parents. In the following sections, the most important findings will be discussed.

As hypothesised, our data suggest improved upper limb function after the first mCIMT training block and retained effects after an 8‐week break, which is consistent with results of previous studies regarding the effectiveness of intensive models of mCIMT in (mostly) older children (Hoare et al., 2019) and in infants and toddlers with unilateral CP (Chamudot et al., 2018; Eliasson et al., 2018; Hwang & Kwon, 2020). The average improvement in Mini‐AHA unit score in our study was almost half of that reported in the study of Chamudot et al. (2018) (Δ8.2 vs. Δ14.5 Mini‐AHA unit score). This disparity may be explained by a higher training intensity (28 hours vs. 56 hours both during eight consecutive weeks), although minimal training intensity to attain improvements for children aged under 18 months is not clear yet (Mailleux et al., 2021).

In line with previous studies that reported improved upper limb function after a home‐based program where therapists used home visits to guide parents (Chamudot et al., 2018; Eliasson et al., 2018), our study found improvements with parents being coached remotely through a video‐coaching approach. Results of previous studies (Ferre et al., 2017; Svensson et al., 2024) using remote coaching of the parents in intensive upper limb interventions are inconsistent. In the study of Ferre et al. (2017), examining the effects of an intensive bimanual training program for older children (aged 2.5–12.5 years), no change in bimanual performance was found, though a recent study showed similar positive effects of a home‐based baby‐CIMT program with remote coaching and with an in‐person approach (Svensson et al., 2024).

The mCIMT training in our study seemed to be most beneficial for children with lower baseline bimanual performance in children aged ≥18 months. This is in accordance with previous findings (Novak & Berry, 2014) in which larger gains in bimanual hand function were assigned to lower baseline function in children mainly aged >2 years. In our study, we noted that some children showed a decline in bimanual performance after the 8‐week break, as was also previously found (DeLuca et al., 2015). This could implicate that these children may not have attained sufficient automaticity in using the affected upper limb, potentially due to suboptimal training duration or breaks after the training block. In our study with a relatively large sample size (*n* = 59), no predictors of treatment response could be identified in children <18 months of age. This was also the case in previous studies with smaller sample sizes (*n* < 20) (Chamudot et al., 2018; Eliasson et al., 2018). Large interindividual differences were noted, consistent with previous findings (Chamudot et al., 2018; Eliasson et al., 2018), indicating the necessity for further research to gain insight into factors influencing treatment response.

Further improvements in bimanual performance were suggested after a second training block, aligning with previous studies that reported positive results from repeated blocks of mCIMT for children older than 11 months (Charles & Gordon, 2007; DeLuca et al., 2015; Grinde et al., 2020). However, in our study, no significant improvements of the second training block were observed in the AHA (children aged ≥18 months). Due to small sample sizes in these sub‐analyses, these results should be interpreted with caution. Additionally, when evaluating the individual change in AHA unit scores, a relatively higher proportion of children showed improvement (≥5 units) after the second training block compared with the first training block, highlighting the clinical relevance of providing a second training block. Given that children experience periods of rapid skill development, a block‐based approach to upper limb intervention may be particularly advantageous. Information about the cumulative effects of second or multiple blocks of CIMT or BiT and the timing of these blocks is needed (Hoare et al., 2019), which would benefit from comparability of the Mini‐AHA and AHA scores. Findings could guide clinician's decision making to optimise outcomes for children with unilateral CP while considering the impact of these interventions on families and healthcare costs (Novak et al., 2013).

The long‐term impact of intensive upper limb training blocks remains uncertain (Hoare et al., 2019; Sakzewski et al., 2014), although the study of Nordstrand et al. (2015) showed promising results. We expect that using a video‐coaching approach that acknowledges the shared expertise of parents and therapists and involves shared decision‐making authority, in line with the principles of Occupational Performance Coaching (OPC) (Graham et al., 2009), may enhance parents' competence in improving their child's bimanual performance. Additionally, we expect that by working with parents as mediators of change (Chien et al., 2021), children's bimanual activity performance in daily life will be enhanced in long‐term. This is supported by statements from parents themselves, as revealed in our interview study (Verhaegh et al., 2022), wherein they indicated that providing the home‐based program themselves was the greatest benefit because they felt educated even beyond the program's scope.

Our study found a similar and relatively high adherence rate (85% vs. 81%) compared with the study by Chamudot et al. (2018). This suggests that the home‐based upper limb training program using a video‐coaching approach is feasible in terms of training intensity. Although it has been suggested that weekly face to face sessions are needed to support parental efficacy for adherence (Harniess et al., 2022), we believe that video coaching can be equally supportive to parents. It must be noted that parents in our study, however, did have face to face contact with a physical therapist at the child's home, not being the therapist who guided the upper limb training program.

### 4.1 Limitations

Our explorative retrospective clinical study, lacking a control group or period, shows weak evidence for the effectiveness of our early upper limb intervention. As we retrospectively collected data in clinical practice in multiple rehabilitation centres, blinded scoring was not possible, which may induce bias. Data collection involved multiple therapists across various rehabilitation centres. Although all were trained in intervention principles and video coaching, differences in clinical protocols may have led to variations in the home‐training program and data quality. However, as the inter‐rater reliability of the AHA is excellent (intra‐class correlation coefficient = 0.97), different raters can be used with a small risk of rater bias (Holmefur et al., 2009). Despite these limitations, our study provides valuable real‐world insights in clinical practice, enhancing ecological validity and supplementing controlled studies.

Because we could not directly compare outcomes measured by the Mini‐AHA and AHA, we had to separately analyse these data resulting in smaller sample sizes, particularly for the second training block. Therefore, we decided to analyse the second block data without considering the different intervention types (mCIMT or BiT). Although we did not expect differences in results (Chamudot et al., 2018), larger randomised controlled trials are needed to compare unimanual and bimanual approaches, as proposed by Boyd et al. (2017).

The generalizability of the second training block results may be limited due to unclear reasons for allocation. Factors such as parental motivation and capability or limited progress during the first block could have influenced this decision.

## 5 CONCLUSIONS AND IMPLICATIONS FOR FUTURE RESEARCH AND CLINICAL PRACTICE

Our data suggest that an 8‐week block of home‐based mCIMT for infants and toddlers with (or at risk of developing) unilateral CP using a video‐coaching approach in clinical practice can improve affected hand use during bimanual activity performance and effects can be retained after an 8‐week break; however, individual results differed. Children aged ≥18 months with lower baseline bimanual activity performance seem to benefit more from the intervention. For children <18 months, no factors associated with treatment response have been identified yet. Our results suggested an improvement in upper limb function after the second training block (mCIMT or BiT) in children <18 months of age and a non‐significant trend for improvement in children aged ≥18 months. Future controlled trials should be conducted in the heterogeneous population of young children with unilateral CP to attain higher levels of evidence regarding the efficacy of early upper limb interventions. These trials could prioritise investigating the intensity and duration of the training blocks, the potential cumulative effects of multiple upper limb training blocks and the duration of the breaks between blocks. Additionally, they should explore unimanual versus bimanual approaches and the effects on brain (re)organisation, as recently proposed (Boyd et al., 2017). Moreover, scores on outcome measures may be less influenced by infant variables such as sleep, hunger or discomfort if the frequency of measurement can be increased. These measurements ideally take place in the infant's home environment, for instance, using accelerometry (Verhage et al., 2023). Findings from controlled trials will guide clinicians in selecting the most effective approach for each individual child, while also considering their impact on family life and healthcare costs.

High adherence rates suggest the program being feasible for parents; however, individual family needs must always be taken into account. A video‐coaching approach may be equally as effective as face‐to‐face therapy sessions in terms of factors influencing parental adherence. Additionally, video coaching may be less intrusive to family life and more flexible than therapist home visits, aligning with the Dutch healthcare strategy. Moreover, video coaching enhances therapists' accessibility to parents, and potentially enhances parents' competence in stimulating their child's bimanual performance as they develop. Adequate functioning digital equipment is crucial for successful implementation of a video‐coaching approach in early upper limb intervention.

## AUTHOR CONTRIBUTIONS

All authors participated in conceptualising the study. Anke P. M. Verhaegh contributed to the methodology, data acquisition, data analysis, drafting the initial manuscript, reviewing and editing. Steven Teerenstra contributed to the methodology, data analysis, reviewing and editing. Maria W. G. Nijhuis‐van der Sanden contributed to the methodology, data analysis, reviewing, editing and supervision. Pauline B. M. Aarts contributed to the methodology, resources, data acquisition, reviewing and editing. Michèl A. A. P. Willemsen contributed to data analysis, reviewing and editing. BG contributed to the methodology, data analysis, reviewing and editing. All authors approved the final manuscript before submission.

## CONFLICT OF INTEREST STATEMENT

The authors have no conflict of interest to declare.

## Data Availability

Data are available from the corresponding author upon reasonable request.
